# Exploration of *N*-Arylsulfonyl-indole-2-carboxamide Derivatives as Novel Fructose-1,6-bisphosphatase Inhibitors by Molecular Simulation

**DOI:** 10.3390/ijms231810259

**Published:** 2022-09-06

**Authors:** Yilan Zhao, Honghao Yang, Fengshou Wu, Xiaogang Luo, Qi Sun, Weiliang Feng, Xiulian Ju, Genyan Liu

**Affiliations:** 1 Hubei Key Laboratory of Novel Reactor and Green Chemical Technology, Key Laboratory for Green Chemical Process of Ministry of Education, School of Chemical Engineering and Pharmacy, Wuhan Institute of Technology, Wuhan 430205, China; 2 School of Materials Science and Engineering, Zhengzhou University, No. 100 Science Avenue, Zhengzhou 450001, China; 3 Key Laboratory of Novel Biomass-Based Environmental and Energy Materials in Petroleum and Chemical Industry, Wuhan Institute of Technology, Wuhan 430205, China

**Keywords:** fructose-1,6-biphosphatase inhibitor, 3D-QSAR, virtual screening, molecular docking, molecular dynamics

## Abstract

A series of *N*-arylsulfonyl-indole-2-carboxamide derivatives have been identified as potent fructose-1,6-bisphosphatase (FBPase) inhibitors (FBPIs) with excellent selectivity for the potential therapy of type II diabetes mellitus. To explore the structure–activity relationships (SARs) and the mechanisms of action of these FBPIs, a systematic computational study was performed in the present study, including three-dimensional quantitative structure–activity relationship (3D-QSAR) modeling, pharmacophore modeling, molecular dynamics (MD), and virtual screening. The constructed 3D-QSAR models exhibited good predictive ability with reasonable parameters using comparative molecular field analysis (*q*^2^ = 0.709, *R*^2^ = 0.979, *r_pre_*^2^ = 0.932) and comparative molecular similarity indices analysis (*q*^2^ = 0.716, *R*^2^ = 0.978, *r_pre_*^2^ = 0.890). Twelve hit compounds were obtained by virtual screening using the best pharmacophore model in combination with molecular dockings. Three compounds with relatively higher docking scores and better ADME properties were then selected for further studies by docking and MD analyses. The docking results revealed that the amino acid residues Met18, Gly21, Gly26, Leu30, and Thr31 at the binding site were of great importance for the effective bindings of these FBPIs. The MD results indicated that the screened compounds **VS01** and **VS02** could bind with FBPase stably as its cognate ligand in dynamic conditions. This work identified several potential FBPIs by modeling studies and might provide important insights into developing novel FBPIs.

## 1. Introduction

Diabetes mellitus (DM) is one of the most common chronic diseases characterized by hyperglycemia in both the fasting and postprandial states of patients [1]. In recent years, the incidence rate of DM has increased exponentially worldwide, which has seriously threatened human lives [2]. Although many effective drugs have been invented in the past hundred years, the prevalence and number of patients with DM have still grown rapidly. According to the International Diabetes Federation report, there were more than 430 million diabetic patients worldwide by 2020, and more than 700 million by 2045 as predicted [3]. DM is divided into three different types: Type I (T1DM), type II (T2DM), and gestational type. As calculated, T2DM accounts for more than 90% of all diabetes [4].

As for the pathogenesis of T2DM, the decrease in glucose utilization and the increase in endogenous glucose production (EGP) lead to an increase in blood glucose levels. EGP in the liver plays an important role in the regulation of blood glucose levels and involves two main processes: Glycogen decomposition and gluconeogenesis (GNG), in which GNG contributes more to the increase in EGP [5,6]. Therefore, the inhibition of GNG is a potential strategy for the treatment of T2DM [7,8,9]. The liver is the major organ that conducts GNG which refers to the process of transforming simple non-carbohydrate precursors such as lactic acid, glycerol, and alanine into glucose or glycogen. Some crucial enzymes are involved in this process, including glucose-6-phosphatase (G6Pase), fructose-1,6-bisphosphatase (FBPase), phosphoenolpyruvate carboxykinase (PEPCK), and pyruvate carboxylase (PC) [10,11]. Liver FBPase has been identified as a promising target for the development of new drugs for T2DM as its inhibition results in smaller adverse interactions than other liver enzymes [12,13,14]. For instance, the inhibition of the enzymes PC and G6Pase could lead to fatty liver and severe hypoglycemia symptoms [15].

Human liver FBPase catalyzes the second step of the GNG pathway by hydrolyzing fructose-1,6-diphosphate (FDP) into fructose-6-phosphate and inorganic phosphate. It is a homo-tetramer, and each monomer contains 337 amino acid residues (36.7 kDa). Two types of binding sites are found in each monomer: An FDP binding site and an adenosine monophosphate (AMP) allosteric binding site [16,17,18]. Besides, a binding pocket was reported to be located at the interface of the two dimers. The AMP binding site has been explored more than the other two sites since it is easier to be bound by small ligands according to the previous reports [19,20].

**Figure 1:**
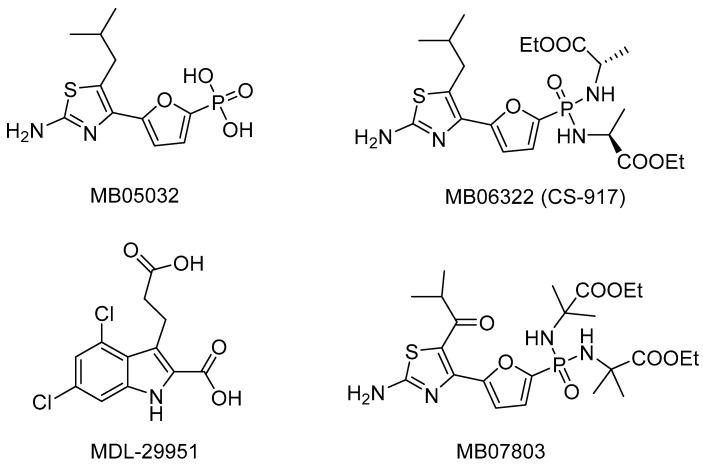
Chemical structures of representative FBPIs.

At present, FBPase inhibitors (FBPIs) could be classified into three types according to their different binding sites: Competitive inhibitors including FDP and endogenous inhibitors that bind to the substrate-binding site, noncompetitive inhibitors that bind to the AMP allosteric site, and anti-competitive inhibitors that bind to another allosteric site at the subunit interface. The reported noncompetitive FBPIs involve purine, benzimidazole, thiazole, and indole derivatives [21]. Representative noncompetitive FBPIs include MB05032 (5-[2-amino-5-(2-methylpropyl)-4-thiazolyl]-2-furanyl]phosphonic acid), MB06322 (l-analine, *N*,*N′*-[[5-[2[amino-5(2-methylpropyl)-4-thiazolyl]-2-furanyl]phosphinylidene]bis-, diethyl ester, CS-917), MB07803 (*N*,*N′*-[[5-[2-amino-5-(2,2-dimethyl-1-oxopropyl)-4-thiazolyl]-2-furanyl]phosphinylidene] diethyl ester), and MDL-29951 (3-(2-carboxyethyl)-4,6-dichloro-1*H*-indole-2-carboxylic acid) (Figure 1). CS-917 and MB07803 were successfully advanced into clinical trials, and CS-917 is the prodrug of MB05032. The phase II study of CS-917 was halted in 2005 due to the toxicity of its metabolite and the adverse effect, which was lactic acidosis in patients when it was administrated with metformin clinically. MB07803 is currently in a phase II clinical trial [22,23,24]. The anti-competitive inhibitors mainly include quinolone and anilinoquinazoline derivatives [25,26].

A novel series of *N*-arylsulfonyl-indole-2-carboxamide derivatives that are modified from a potent FBPI MDL-29951 has recently been identified as noncompetitive FBPIs with powerful inhibitory activity [27]. To better understand their structure–activity relationships (SARs) and mechanism of action with FBPase, a systematical in silico study, including 3D-QSAR modeling, pharmacophore modeling, molecular docking, and molecular dynamics (MD) simulations, was carried out in this study. To explore novel FBPI hit compounds, virtual screening was then performed using the constructed pharmacophore model, molecular dockings, and ADME predictions. MD simulations were also applied to validate the stability of the screened hits in the active site of FBPase. This study might provide important information on the design of novel FBPIs and could nominate more potential FBPI candidates for future studies.

## 2. Results and Discussion

### 2.1. 3D-QSAR Model

**Figure 2:**
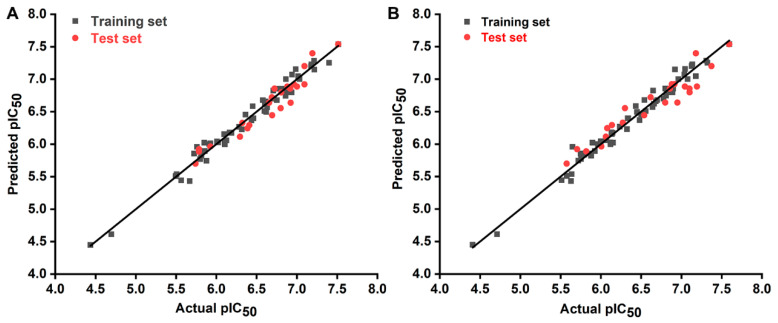
Scatter plots of actual vs. predicted pIC_50_ values for the training (black squares) and test set (red dots) compounds by the CoMFA (**A**) and CoMSIA (**B**) models.

A set of 85 *N*-arylsulfonyl-indole-2-carboxamide derivatives as FBPIs (Table S1) was selected for 3D-QSAR modeling to investigate their inhibitory activity and structural features. The statistical results of the internal and external validations of the constructed comparative molecular field analysis (CoMFA) and comparative molecular similarity indices analysis (CoMSIA) models are summarized in Table 1 and Table 2, respectively. In the CoMFA model, the obtained parameters (*q*^2^ = 0.709, *R*^2^ = 0.979, *F* = 391.215, *r_pre_*^2^ = 0.932, RMSE = 0.136, and MAE = 0.227) suggested that this model exhibited good predictive ability and stability. The contribution rates of steric (S) and electrostatic (E) fields for this model were 72.0% and 28.0%, respectively, indicating that substituent volumes might be the dominant factor for the inhibitory activity of these sulfonamide FBPIs compared to the electrostatic effect. Based on different combinations of S, E, hydrophobic (H), H-bond donor (D), and H-bond acceptor (A) fields, fourteen CoMSIA models were generated, and the contributions of different fields reflected their influence on the activity of compounds. The CoMSIA-SEDA model was selected for further analysis due to its relatively better predictive capacity with the following parameters: *q*^2^ = 0.716, *R*^2^ = 0.978, *F* = 255.130, *r_pre_*^2^ = 0.8898, RMSE = 0.1730, and MAE = 0.2227. Four force fields that constitute the CoMSIA-SEDA model had different influences on the activity of compounds, and the contribution rates of S, E, D, and A fields were 21.0%, 45.3%, 16.4%, and 17.3%, respectively. These data indicated that the E field had a relatively higher influence on the activity in this model. As shown in Figure 2, the actual and predicted activity of all compounds were near the trend line, indicating the rationality and reliability of the 3D-QSAR models.

### 2.2. Contour Map Analysis

**Figure 3:**
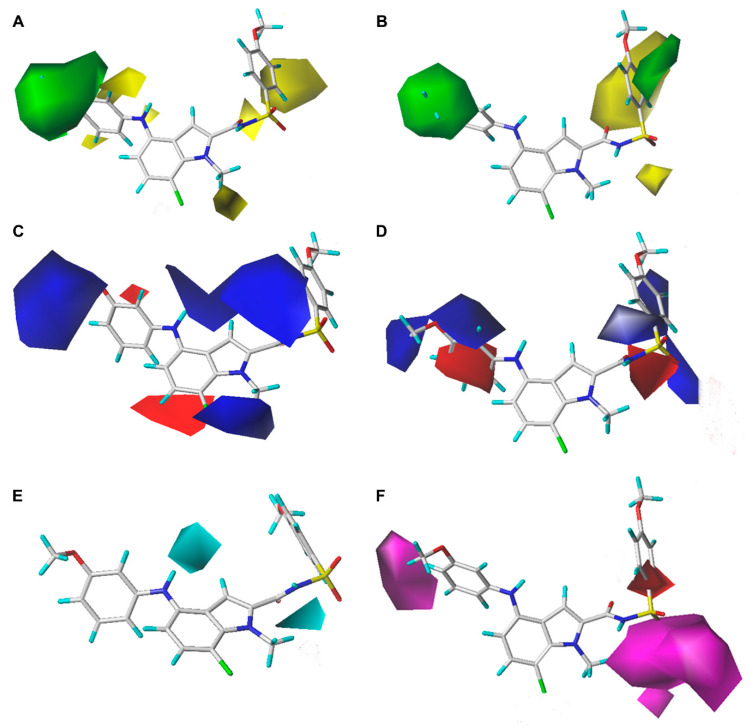
Contour maps of the CoMFA and CoMSIA models using compound **75** as a reference. (**A**) The steric field contour map of the CoMFA model. (**B**) The steric field contour map of the CoMSIA model. (**C**) The electrostatic field contour map of the CoMFA model. (**D**) The electrostatic field contour map of the CoMSIA model. (**E**) The H-bond donor field contour map of the CoMSIA model. (**F**) The H-bond acceptor field contour map of the CoMSIA model.

The CoMFA and CoMSIA contour maps are shown in Figure 3 using compound **75** as a reference and provide useful information about the SARs of these sulfonamide FBPIs. In the steric contour maps of the CoMFA and CoMSIA models (Figure 3A,B), the green area indicates that the introduction of bulky groups into this position might be beneficial to the activity, whereas the yellow area represents the idea that bulky groups are not suitable for increasing the activity. Large green contours were found to cover the whole R^6^-substituted phenyl ring of compound **75** in both CoMFA and CoMSIA models, indicating that bulky groups at the C^4^-position of the indole ring might be favorable for activity. This could be proved by the fact that the inhibitory activities of compounds **17**, **18**, **25**, and **14** with *N*-aryl groups at the C^4^-position were higher than those of the corresponding compounds **76**, **77**, **80**, and **85** with small groups in the same place, respectively. Another small green contour was close to the R^1^ position in the CoMSIA model, implying that bulky groups there might be beneficial for increasing the activity. It could be verified by the activity orders of **3** (R^1^-3-methoxyphenyl) > **2** (R^1^-Ph) > **1** (R^1^-cPr) and **12** (R^1^-naphthalen-2-yl) > **11** (R^1^-thiophen-2-yl) > **13** (R^1^-Ph). The large yellow areas were distributed near the amide bond as a linker in the middle of the skeleton in both CoMFA and CoMSIA models. Another yellow contour was found around the R^6^ position of the phenyl ring in the CoMFA model, suggesting that bulky substituents there were not beneficial to activity, which could be supported by the following activity order: **50** (R^6^-acetamido) > **51** (R^6^-3,5-dimethoxy) > **52** (R^6^-3,4,5-trimethoxy).

The electrostatic contour maps are shown in Figure 3C,D. The blue areas indicated regions where more positively charged substituents are favored, and the red areas suggested regions where more negatively charged substituents are favored. In both CoMFA and CoMSIA models, two large blue contours were located near the *N*-aryl moiety of the indole ring and the sulfonamide group, respectively, indicating that positively charged groups there might be helpful for the binding affinity. Another medium blue contour was found around the R^2^ and R^5^ positions of the indole ring in the CoMFA model, which could be verified by the following activity orders: **3** (R^2^-H) > **46** (R^2^-Et) and **54** (R^5^-NO_2_) > **56** (R^5^-Cl). The red contours were mostly found in the R^6^ position of the phenyl ring in the CoMSIA model, as observed in the activity orders: **3** (R^6^-3-OMe) > **17** (R^6^-3-Me) and **31** (R^6^-4-Cl-3-OMe) > **28** (R^6^-dimethoxy). Another red contour was close to the R^3^ position of the indole ring in the CoMFA model, which could be supported by the activity orders: **34** (R^3^-F) > **35** (R^3^-Cl) and **38** (R^3^-F) > **37** (R^3^-H).

As exhibited in Figure 3E,F, the H-bond donor and H-bond acceptor contour maps revealed the favorable locations where H-bond donors and H-bond acceptors might enhance inhibitory activity. Two cyan contours were exactly near the two imino groups of compound **75**, suggesting that imino groups as H-bond donors at this position might be useful to improve the inhibitory activity. Two large magenta contours were close to the R^6^ position of the phenyl ring and the sulfonamide group connected to the indole ring, respectively, indicating that H-bond acceptors there might contribute to the activity. This could be corroborated by the following activity orders: **16** (R^6^-3-OCF_2_H > **17** (R^6^-3-Me), **4** (R^6^-3-OMe) > **18** (R^6^-3-Et), and **54** (R^6^-OMe) > **48** (R^6^-Me).

In summary, the following structural features are considered to be conducive to the inhibitory activity of FBPIs: (a) Positively charged and/or bulky groups at the R^1^ position; (b) positively charged and/or small groups at the R^2^ and R^5^ positions; (c) negatively charged and/or small groups at the R^6^ position of the phenyl ring; and (d) H-bond donors in the linker moiety and H-bond acceptors at the R^6^ position of the phenyl ring.

### 2.3. Pharmacophore Modeling

To further explore the key structural features of these FBPIs, a cluster of eight compounds with diverse structures and relatively high activities were selected to generate pharmacophore models. The top ten models were listed in Table 3. MODEL _04 was comprehensively considered to be the best model with the following parameters: SPECIFICITY = 4.327, N_HITS = 8, FEATS = 9, PATERO = 0, ENERGY = 68.89, STERICS = 2241.9, HBOND = 716.2, and MOL_QRY = 184.60. The model was then validated by the decoy set method to determine its quality and reliability. The calculated validation parameters were EF = 13.741 (EF > 1) and GH = 0.675 (0.6 < GH < 0.8), suggesting that the best model was able to distinguish active compounds from inactive compounds and could be used in the following experiment on virtual screening.

**Figure 4:**
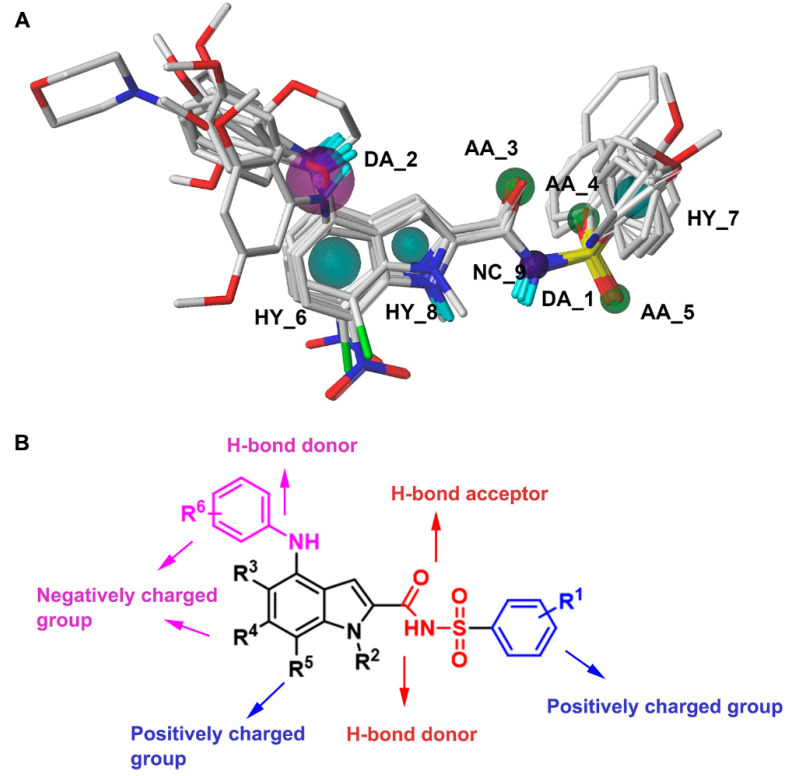
The constructed pharmacophore model (**A**) and the summarized SAR (**B**) of *N*-arylsulfonyl-indole-2-carboxamide derivatives as FBPIs.

Moreover, this model has nine pharmacophore features, including three hydrophobic centers (HYs), two H-bond donors, three H-bond acceptors, and one negative center (NC). The hydrophobic centers were distributed in the indole ring and the aromatic ring, two H-bond donors were distributed in the imino group, three H-bond acceptors were distributed in the carbonyl group and the sulfonyl group, and the negative center was distributed in the imino group of the sulfonamide moiety. The structural features from the best pharmacophore model in combination with those from the 3D-QSAR analysis are summarized in Figure 4.

### 2.4. Virtual Screening

**Figure 5:**
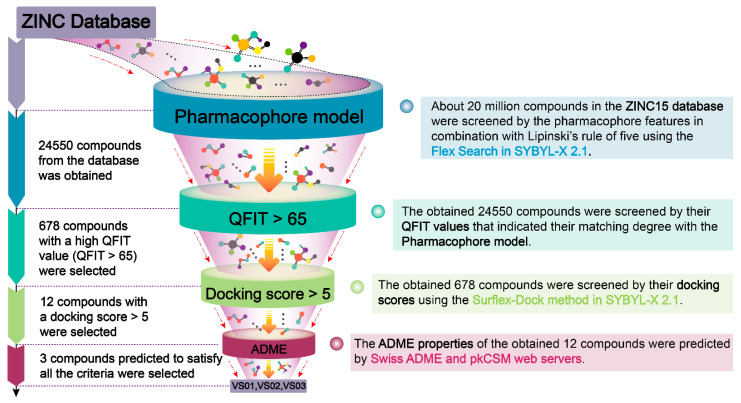
Four rounds of screenings in the virtual screening process.

The virtual screening process is displayed in Figure 5 and mainly comprises four rounds of screenings. In the first-round screening, the best pharmacophore model (MODEL_04) was primarily transformed into a UNITY search query for screening against the ZINC15 database that contained approximately 20 million compounds. To assess the drug-like properties of the compounds in the database and whether they meet the pharmacophore features concluded from the best pharmacophore model, the ZINC15 database was screened by the Flex search query with the specified query option of using Lipinski’s rule of five. Through this step, 24,450 compounds were obtained in the UNITY search.

The QFIT value reflects the consistency of compound structures with the pharmacophore features. A high QFIT value indicates a high matching degree of a compound with the best pharmacophore model. Thus, in the second-round screening, a high QFIT standard (QFIT > 65) was applied, and 678 compounds were selected in this step. Then, the third-round screening was performed using molecular docking. The screened 678 compounds from the last step were docked into FBPase by the Surflex-Dock method. The docking score > 5 was the range of the docking scores of these highly active *N*-arylsulfonyl-indole-2-carboxamide derivatives (Table S1) and was set as the screening parameter, which might help select compounds with ideal activity. Twelve compounds with a docking score > 5 were obtained in this step. Their chemical structures and docking scores are listed in Table S2. These compounds all contained a sulfonamide group as a linker moiety of two aromatic rings. The indole rings of several compounds were connected to the sulfonamide groups, and the substituents on the benzene rings were negatively charged and/or small groups. These structural features were consistent with the results of the SAR analysis.

Next, in the fourth-round screening, the two web servers SwissADME and pkCSM were used in a complementary way to predict the ADME properties of these twelve compounds to evaluate whether they could be further developed as drug candidates. The physiochemical properties, water solubility, lipophilicity, gastrointestinal (GI) absorption, and the blood–brain barrier (BBB) penetration of these compounds were predicted by SwissADME. The drug-like properties and synthetic accessibility (SA) were predicted by pkCSM. The selected 12 compounds were subjected to the prediction of their pharmacokinetic properties, and only three compounds **VS01** (ZINC code: 15733809), **VS02** (ZINC code: 02961023), and **VS03** (ZINC code: 02961075) (Table 4) satisfied all the following conditions: 150 < molecular weight (*M*_W_) < 500 g/mol; 20 < total polar surface area (TPSA) < 130 Å^2^; H-bond donors < 5; H-bond acceptors < 10; no violation in drug-like properties [28]. Besides, the screened three compounds were predicted to have good lipophilicity and high GI absorption. They could not penetrate the BBB and could be easily synthesized with low synthetic accessibility (SA) scores [29]. The prediction data of compounds **75**, **VS01**, **VS02**, and **VS03** are summarized in Table S3. The other compounds in Table S2 were eliminated due to their poor lipophilicity, low GI absorption, or some violations in drug-like properties.

### 2.5. Molecular Docking

**Figure 6:**
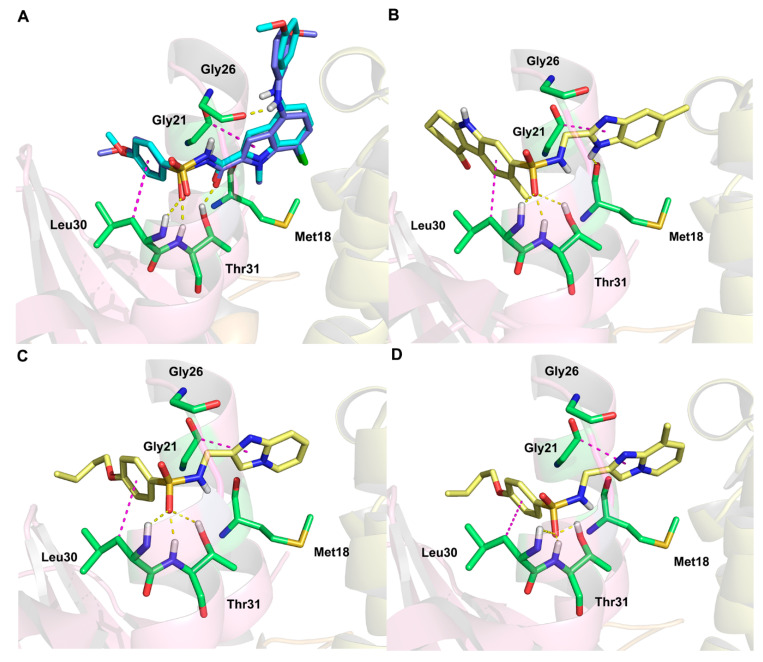
The docking results of compound **75** (**A**, superimposition by its cognate structure), **VS01** (**B**), **VS02** (**C**), and **VS03** (**D**). The redocked ligand, co-crystallized ligand, H-bonds, and hydrophobic interactions are shown as blue sticks, cyan sticks, yellow dashes, and magenta dashes, respectively.

The crystal structure of FBPase in complex with compound **75** was resolved in 2020 (PDB code: 6LW2) [27]. In this work, the co-crystallized compound **75** was extracted and then redocked into FBPase by the Surflex-Dock Geom module in SYBYL-X 2.1 software to validate the docking method. The root mean square deviation (RMSD) between these two docking conformations was 0.62 Å, which was less than 2.0 Å, demonstrating that the docking method was reliable. The 86 *N*-arylsulfonyl-indole-2-carboxamide derivatives were docked into the AMP site of FBPase using the same method. The docking scores of these compounds were found to be basically consistent with their actual activities. The initial and redocked conformations of compound **75** are shown in Figure 6A. Five H-bonds were formed between compound **75** and residues Gly26 (Gly26-O…HN, 2.1 Å), Leu30 (Leu30-NH…O=S, 2.3 Å), and Thr31 (Thr31-NH…O=S, 1.9 Å; Thr31-OH…O=C, 1.7 Å, Thr31-OH…O=S, 2.8 Å). These H-bonds might be significant for the stable binding of compound **75** in FBPase. Besides, two hydrophobic interactions existed between the indole ring and phenyl ring of **75** and the alkyl chains of Gly21 and Leu30, respectively, further enhancing the protein–ligand binding. These interactions were supported by previous reports [18,30], which also confirmed that this docking method was accurate. Then the screened compounds were docked into the AMP site of FBPase to explore their binding modes and key interactions of these FBPIs with FBPase. The structure of FBPase and the AMP binding site are shown in Figure S1. As shown in Figure 6, the FBPIs were inserted into the same binding pocket composed of amino acid residues Met18, Gly21, Gly26, Leu30, and Thr31. Their binding modes were basically consistent.

As shown in Figure 6B, **VS01** formed H-bonds with Leu30 (Leu30-NH…O=S, 2.2 Å) and Thr31 (Thr31-NH…O=S, 1.8 Å; Thr31-OH…O=S, 2.8 Å). Another different H-bond existed between **VS01** and Met18 (Met18-O…HN, 3.1 Å). Two hydrophobic interactions were also found between the indole ring and phenyl ring of **VS01** and the alkyl chains of Gly21 and Leu30, respectively. Compared to **VS01**, **VS02** (Figure 6C) formed equivalent H-bond interactions with Leu30 (Leu30-NH…O=S, 2.2 Å) and Thr31 (Thr31-NH…O=S, 1.8 Å; Thr31-OH…O=S, 2.8 Å). **VS03** (Figure 6D) formed H-bond interactions with Leu30 (Leu30-NH…O=S, 3.6 Å) and Thr31 (Thr31-NH…O=S, 1.3 Å; Thr31-OH…O=S, 1.6 Å). The H-bond between the sulfonyl group of **VS03** and the imino group of Leu30 with a length of 3.6 Å was longer than those of **VS01** and **VS02**, which might explain the weaker H-bond strength and its lower docking score. The same hydrophobic interactions were found between the aromatic rings of both **VS02** and **VS03** and the residues Gly21 and Leu30. The above-mentioned results revealed that some key amino acid residues in the binding pocket, including Gly26, Leu30, and Thr31, were crucial for the formation of H-bond interactions with these FBPIs. The hydrophobic interactions also mattered to the effective binding between these FBPIs and the residues. Besides, Met18 was found to be a noticeable residue that formed an H-bond with **VS01** in addition to those with Leu30 and Thr31, which might explain its higher docking score than **VS02** and **VS03**. The docking results showed that these screened hits could bind to the protein effectively and might provide some reference for developing novel FBPIs.

### 2.6. MD Simulation

Molecular docking and MD simulation have been regarded as two complementary strategies to identify the molecular interactions between ligands and proteins [31]. By performing MD simulations, the rationality of docking results could be validated, and the dynamic behavior of molecular arrangements could be probed at different timescales, allowing the identification of conformational changes in ligands at the active site of the protein [32]. The tetrameric structure of FBPase was used in the MD simulations. To examine the stability of the screened hits in complex with FBPase in a dynamic environment, 50 ns MD simulations were performed on the following complexes: FBPase without am inhibitor, FBPase-**75**, FBPase-**86**, FBPase-**VS01**, and FBPase-**VS02**. Compound **86** was used as a negative control to validate that the MD simulations could identify the compounds with good binding affinities. Since **VS01** had an apparently higher docking score and **VS02** had a slightly lower score than compounds **75**, **VS01** and **VS02**, instead of **VS03**, were tested by MD simulations on their binding stability with FBPase.

The 50 ns simulation of the FBPase-**75** complex was extended to 150 ns, and the results are displayed in Figure S2. The results of 150 ns were very similar to those of the 50 ns simulation, indicating that 50 ns simulation might be enough for the FBPase protein. Thus, we finally chose to conduct 50 ns MD simulations for these systems. In order to improve the quality of the MD simulations, triplicate runs of 50 ns were performed on the four complexes: FBPase-**75**, FBPase-**86**, FBPase-**VS01**, and FBPase-**VS02**, respectively. The obtained results of the triplicate runs for each system are demonstrated in Figures S3–S7. Some dynamic parameters, including the RMSD, root mean square fluctuation (RMSF), and gyration radius (R_g_), were calculated during the simulations to evaluate the binding stability of these ligand–protein complexes. The RMSD value revealed the changes in the distance of atoms from their original positions, which reflected the stability of different objects in the system [33]. The RMSF values of ligands in the complex reflect the change in the position of each atom. For different atoms, a higher RMSF value implies a greater position change and greater flexibility in the binding pocket during the simulation. The overall fluctuation trend reflects the binding stability of the ligand. The RMSF value of the chain residues was adopted to measure the degree of amino acid residue fluctuation and flexibility in the protein. The R_g_ value was applied to illustrate the tightness of the protein throughout the simulation. As demonstrated in Figure S5, the third simulation (MD3) of each system with the most stable trajectories. which could be exhibited by the relatively lowest RMSF value of the ligand among the triplicate simulations for each system, was chosen for further analysis.

**Figure 7:**
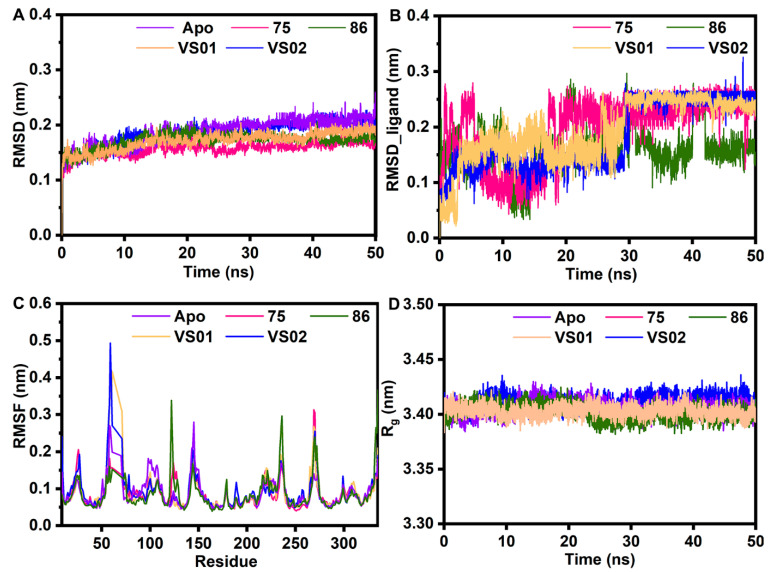
The MD results of the five complex systems: apo FBPase (purple), FBPase-**75** (magenta), FBPase-**86** (green), FBPase-**VS01** (orange), and FBPase-**VS02** (blue). (**A**) The RMSD of the backbone atoms of the proteins. (**B**) The RMSD of the ligands (**75**, **86**, **VS01**, **VS02**). (**C**) The RMSF of the Chain A residues. (**D**) The R_g_ of the proteins.

As shown in Figure 7A, the RMSD values of the protein backbone in these systems had fluctuations in the first 30 ns and became stable at 0.2 nm after 30 ns. The RMSD values of the three ligands reached convergence after 30 ns. The RMSD values of **VS01** and **VS02** showed similar fluctuations in the first 30 ns and were stable after 30 ns. As displayed in Figure 7B, the RMSD value of compound **75** had greater fluctuations in the first 30 ns, implying that it underwent bigger conformational changes in this period than **VS01** and **VS02**. The RMSD values of compound **86** exhibited continuous fluctuations during the whole simulation, which indicated that the system of compound **86** in the complex with FBPase was less stable than the others. What is more, **VS01** reached equilibrium at the lowest RMSD value of 0.24 nm among the three systems, which indicated that **VS01** might bind more stably to the receptor than the other two ligands.

As shown in Figure 7C, the fluctuation magnitude of the Chain A residues in the three systems was approximately the same, suggesting that the ligands in these systems had similar binding patterns with FBPase. The key residues from Met18 to Thr31 at the binding pocket exhibited low flexibility with RMSF values of less than 0.2 nm. As displayed in Figure 7D, the R_g_ values maintained a stable state between 3.38 and 3.43 nm without significant changes in the five systems. Comparing the results of the apo and inhibitor-bound FBPase complexes, both the RMSD and the R_g_ of the protein backbone reached equilibrium at relatively lower values in the inhibitor-bound FBPase systems than in the apo FBPase system, indicating that the binding of inhibitors enhanced the stability of the systems.

**Figure 8:**
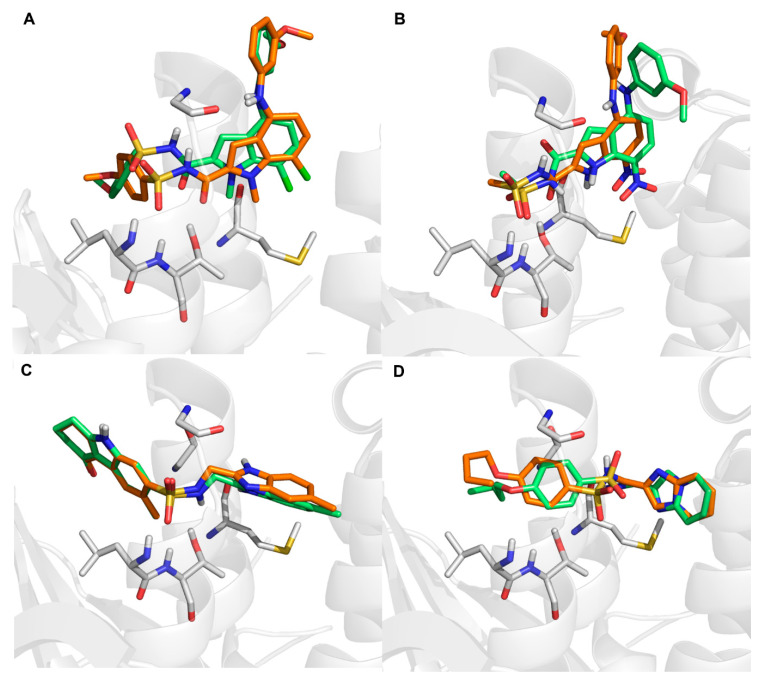
The initial (green sticks) and final (orange sticks) conformations of compounds **75** (**A**), compound **86** (**B**), **VS01**(**C**), and **VS02** (**D**) in 50 ns MD simulations.

The initial and final binding conformations of the compounds were retrieved from the MD trajectories at 0 ns and 50 ns, respectively. As shown in Figure 8, compounds **75**, **VS01**, and **VS02** displayed a small conformational change with slight rotations of some bonds. By contrast, compound **86** exhibited a relatively conformational change during the MD simulation, which indicated the less stable binding of compound **86** with FBPase compared with the other three compounds.

The binding free energies of the three systems were calculated by the MM-PBSA method to analyze the binding affinities of these compounds. As shown in Table 5, the binding free energies of compounds **75**, **86**, **VS01**, and **VS02** in FBPase were −82.73, −42.87, −107.42, and −97.40 kJ/mol, respectively. The results implied that **VS01** and **VS02** might interact with FBPase with stronger binding affinities than compounds **75** and **86**, and **VS01** might have the strongest binding ability. The van der Waals energy of **VS01** was significantly lower than those of compounds **75**, **86**, and **VS02**, which might result in their more favorable binding free energies. Besides, the polar solvation energy of compound **75** was relatively high, which was unfavorable for the total binding free energy. Hence, the van der Waals and polar solvation energies made great contributions to the binding affinities of these compounds. According to these analyses, the screened hits **VS01** and **VS02** might be potential candidates for the development of FBPIs.

## 3. Materials and Methods

### 3.1. Dataset and Optimization

The chemical structures and pIC_50_ (-logIC_50_) values of 85 *N*-arylsulfonyl-indole-2-carboxamides as novel FBPIs are shown in Table S1 [27]. Compounds **75** (IC_50_ = 0.029 μM) and **86** (IC_50_ > 50 μM) were used as positive and negative controls in this study, respectively. The 3D structures were constructed by the sketch module of SYBYL-X 2.1 software (Tripos Inc., St. Louis, MO, USA) and were then optimized by the Tripos force field using Gasteiger–Hückel charges with a gradient of 0.0005 kcal/(mol·Å) and a maximum iteration number of 10,000. The other parameters were set as default [34]. The optimized 85 compounds were stored in a new database.

### 3.2. 3D-QSAR Study

**Figure 9:**
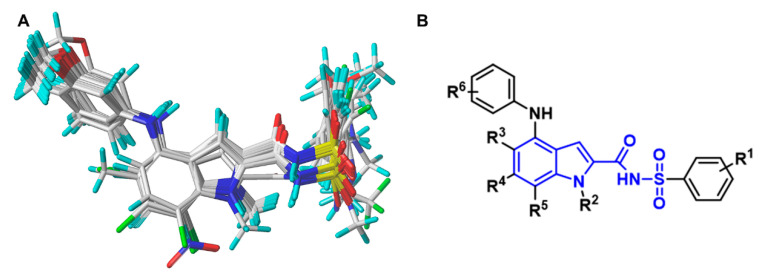
Molecular alignment of the 3D-QSAR models. (**A**) The alignment result of all the training set molecules. (**B**) The common skeleton (blue) of *N*-arylsulfonyl-indole-2-carboxamides used for the alignment.

The dataset of the 85 *N*-arylsulfonyl-indole-2-carboxamides was subjected to molecular alignment using compound **75** as a template as it had the second-lowest IC_50_ value and the best bioavailability among all the molecules. The molecular alignment of the database was conducted by the alignment tool in SYBYL-X 2.1 software. By selecting the common skeleton (shown in blue color) of these structures on the template molecule, the molecules were superimposed onto the selected common skeleton automatically. The alignment result was displayed in Figure 9. The 85 compounds in the aligned database were then randomly divided into a training set of 61 compounds and a test set of 24 compounds. The training set molecules were utilized to generate 3D-QSAR models, and the test set molecules were used to further validate the prediction ability of the constructed models.

In this study, the 3D-QSAR models were constructed using the CoMFA and CoMSIA methods by the QSAR analysis tool in SYBYL-X 2.1 software [35]. First, the CoMFA and CoMSIA properties of the training set were calculated, respectively. The CoMFA model involves two different fields: Steric and electrostatic fields. The CoMSIA model involves five different fields, including steric, electrostatic, hydrophobic, H-bond donor, and H-bond acceptor fields, and the CoMSIA model could be generated using different combinations of these five fields. The constructed CoMFA and CoMSIA models were transformed to contour maps, which were used for the SAR analysis. To accomplish the constructions of the CoMFA and CoMSIA models, the training set was subjected to the partial least squares (PLS) regression analysis. The optimal number of compounds (ONC) was first set to 10 as a test value in the Leave-one-out (LOO) cross-validation. Then the actual ONC and cross-validated coefficient (*q*^2^) were calculated by the LOO cross-validation. *q*^2^ is defined by the following formula:

(1) $$q^{2}=1-\frac{\sum {\left(r_{pre}-r_{exp}\right)}^{2}}{\sum {\left(r_{exp}-r_{mean}\right)}^{2}}$$

in which *r_exp_*, *r_mean_*, and *r_pre_*^2^ represent the experimental, mean, and predicted pIC_50_ values, respectively.

Then the ONC was set to the actual ONC, and the generated models were validated by the calculated non-cross-validation parameters, including the squared correlation coefficient (*R*^2^), standard error of estimate (SEE), and Fischer test value (*F*). The above-mentioned parameters were internal validation parameters. A reliable CoMFA or CoMSIA model should satisfy the following internal parameter ranges: *q*^2^ > 0.5, *R*^2^ > 0.6, SEE << 1, and *F* > 100 [36]. Subsequently, external validation was conducted to further examine the quality of the constructed models. The activities of the test set compounds were predicted by the constructed models, and the predicted pIC_50_ and actual pIC_50_ values of the test set compounds were used to perform the linear regression analysis and generate scatter plots. The external validation parameters mainly involve *k*, *k′*, *r*^2^, *r_0_*^2^, *r_0_′*^2^, *r_m_*^2^, *r_m_′*^2^, Δ*r_m_*^2^, $\bar{r_{m}{}^{2}}$, *r_pre_*^2^ , and root mean square error (RMSE), which were calculated in our previous reports [37,38,39]. The constructed 3D-QSAR models are judged to have good prediction ability by the following requirements: (*r*^2^ − *r_0_*^2^)/*r*^2^ < 0.1, 0.85 ≤ *k* (or *k′*) ≤ 1.15, Δ*r_m_*^2^ < 0.2, $\bar{r_{m}{}^{2}}$ > 0.5, and *r_pre_*^2^ > 0.5 [40].

### 3.3. Pharmacophore Modeling

The pharmacophore model was constructed using the Genetic Algorithm with Linear Assignment of Hypermolecular Alignment of Database (GALAHAD) module of SYBYL-X 2.1 software (Tripos Inc., St. Louis, MO, USA), and 20 models with varied parameters including SPECIFICITY, N_HITS, STERICS, and ENERGY were firstly generated. The pharmacophore model suitable for screening should basically meet the following requirements: SPECIFICITY > 4; N_HITS equals the number of compounds used for the construction; relatively low energy indicating stability. After comprehensively evaluating the parameters generated by the 20 models, the model with the most favorable values was chosen for further analysis. A decoy set method was then applied to evaluate the quality of the model. The decoy set in this study was composed of 1519 compounds downloaded from the DUD-E database (http://dud.docking.org/, accessed on 16 April 2021) [41] and 77 active compounds from Table S1 except for the compounds used for the construction of the model. The enrichment factor (EF) and Güner-Henry (GH) Score were used to judge the reliability of the model. When the values of EF and GH Score meet *EF* > 1, 0.6 < *GH* < 1, the model could be deemed viable for further study [42,43]. EF and GH are defined by the following formulas (2) and (3), in which *H_a_*, *H_t_*, *A*, and *D* represent the number of true positive compounds in the hit list generated by the pharmacophore-based screening, the number of all compounds in the hit list, the number of true positive compounds in the database, and the number of all compounds in the database, respectively.

(2) $$EF=\frac{H_{a}/H_{t}}{A/D}$$

(3) $$GH=\left[\frac{H_{a}\left(3A+H_{t}\right)}{4H_{t}A}\right]\left(1-\frac{H_{t}-H_{t}}{D-A}\right)$$

### 3.4. Molecular Docking

Molecular docking is an in silico method employed to foresee the binding modes of molecules with a receptor [44]. In this study, the crystal structure of human liver FBPase (PDB code: 6LW2) in a complex with compound **75** was used for the dockings. This structure showed that *N*-arylsulfonyl-indole-2-carboxamides bind to the AMP allosteric site of FBPase. Some pretreatments of the protein were performed before the dockings, including hydrogen addition, charge addition, repair of side chains, water removal, and the extraction of co-crystallized ligand. The docking site was generated using the ligand-based mode with a default threshold of 0.5 Å by the Surflex-Dock Geom module in SYBYL-X 2.1 software. The co-crystallized ligand of FBPase was first redocked into the binding pocket to examine whether the docking method was reasonable. The conformational distance between the redocked and original ligands was evaluated by the RMSD values. RMSD < 2.0 Å is regarded as a standard of the dependability of the docking method [45]. The selected FBPIs were then docked into the binding site using the same docking method, and a total of 20 different conformations were generated for each compound. The docking conformation with the highest total score was picked for further analysis.

### 3.5. Virtual Screening

In this study, the screening process was virtual and mainly involved four rounds of screenings. The first-round screening aimed to select the compounds that matched the pharmacophore features of the best pharmacophore model and had drug-like properties. The best pharmacophore model (MODEL_04) was first converted into a UNITY search query by the UNITY search system in SYBYL-X 2.1 software. The screening was performed against the ZINC15 purchasable database (http://zinc.docking.org, accessed on 2 June 2021). In order to eliminate the molecules inappropriate for being developed as drug candidates and narrow the scope of screening, Lipinski’s rule of five was used for the preliminary screening against the database. To start the search, the query type was set as a Flex Search and the query option was specified by activating the option of Lipinski’s rule of five. Then the molecules in the database that mismatched the pharmacophore model and failed to obey Lipinski’s rule of five were filtered out simultaneously. The compounds that fitted the pharmacophore model and satisfied Lipinski’s rule of five were retrieved from the database.

After the first round of screening, a column of QFIT parameters was loaded with the screened compounds. QFIT is a value between 0 and 100, where 100 is the best. It represents how close the ligand atoms of the compounds match the query target coordinates. The QFIT value is not specified and is generally set to a high value (usually more than 50) to reduce the number of hits and to ensure the screened compounds have a higher matching degree with the pharmacophore characteristics [46]. In this study, the minimum standard of QFIT was first set to 50, and 5728 compounds with QFIT > 50 were obtained. In order to improve the screening efficiency and further exclude compounds, the minimum QFIT value of these compounds was raised to 65. Therefore, the compounds with a high QFIT value (QFIT > 65) were finally selected by the second-round screening. In the third-round screening, the compounds with QFIT > 65 were docked into FBPase by the Surflex-Dock method. The docking scores of the 85 compounds (Table S1) with IC_50_ values < 1 μM all yielded docking scores of more than 5. The compounds with docking scores > 5 were selected for potentially strong activities. Finally, in the fourth-round screening, the selected compounds were subjected to the predictions of their physiochemical and pharmacokinetic properties by the two web servers: SwissADME (http://www.swissadme.ch, accessed on 2 June 2021) and pkCSM (http://structure.bioc.cam.ac.uk/pkcsm, accessed on 16 June 2021). The compounds that obey all the following basic principles, including appropriate MW and TPSA, no more than five H-bond donors and ten H-bond acceptors, and no Lipinski violation, were selected for further study [47,48].

The hit compounds with desirable pharmacophore compatibility, preferable docking scores, and ideal prediction results of ADME properties were further studied in their mechanism of action by molecular dockings and their binding stability with FBPase by MD simulations. The molecular dockings helped identify the reasonable binding conformation of each screened hit by comparing it with the co-crystallized compound **75**. The key residues in the binding site of FBPase and the interactions that might influence the activities of the compounds were also revealed, which might explain the fundamental reason for the different activities. The binding stability of each compound was reflected by the fluctuation of various parameters in the MD simulations and the conformation comparisons of the compound before and after the simulations. Smaller changes indicated a more stable binding of the compound with FBPase.

### 3.6. MD Simulation

To examine the dynamic stability of the screened hits in the receptor, 50 ns MD simulations were performed on the docked conformers in complex with URAT1 by GROMACS 2019.5 software (Uppsala University, Stockholm University, and the Royal Institute of Technology, Sweden). The pdb2gmx tool was subjected to generating the topology files of the protein under the AMBER99SB force field [49]. The ACPYPE tool was used to generate the topology files of the ligands optimized by the Tripos force field in SYBYL-X 2.1 software before the MD simulations. The complexes were then placed in a cubic box with a radius of 12 Å filled with water molecules, and six chloride ions were added to neutralize the systems. Then the energy minimizations of the systems were performed by the steepest gradient descent method to converge at an energy tolerance of 10 kJ/mol [50,51]. After that, the NVT and NPT equilibrations of 100 ps were reached at the temperature of 300 K and under the pressure of 1 atm. After MD simulations, some parameters including RMSD, RMSF, and R_g_ were measured using inbuilt commands to analyze the stability changes in the protein during the simulation process. In addition, the binding free energy of each ligand in the protein was calculated using the molecular mechanics Poisson–Boltzmann surface area (MM-PBSA) method from the equilibrium trajectory of the last 5 ns in the whole MD simulation, which contained 50,000 snapshots [52,53,54]. Δ*G_bind_* is calculated according to the following equation:

(4) $$\Delta G_{bind}=G_{complex}-G_{receptor}-G_{ligand}$$

where *G_complex_*, *G_receptor_*, and *G_ligand_* are the calculated Gibb’s free energy of the ligand-receptor, the receptor, and the ligand, respectively. The free energies for each of these terms were estimated as the sum of the four terms below:

(5) $$G=E_{MM}+G_{psolv}+G_{npsolv}-TS_{n\mathrm{mod}e}$$

where *E_MM_* is the molecular mechanics energy calculated as the sum of the molecular internal energy, the electrostatic, and van der Waals interactions. *G_psolv_* and *G_npsolv_* are the polar and nonpolar contributions to the molecular solvation energy, respectively. T is the absolute temperature, and *S* is the entropy of the molecule estimated by normal-mode analysis.

## 4. Conclusions

In this study, a series of *N*-arylsulfonyl-indole-2-carboxamide derivatives as novel FBPIs were studied to identify their SARs and mechanism of action by systematically computational studies. The SARs of these compounds were revealed by the constructed 3D-QSAR models and the pharmacophore model from the perspective of molecular modeling. The sulfonamide group as a linker could serve as an H-bond donor and/or receptor. The negatively charged and/or small substituents on the benzene ring and the positively charged and/or small substituents on the indole ring might be helpful for the inhibitory activity. The indole moiety and the positively charged and/or bulky substituents connected to the linker could form hydrophobic interactions with relative amino acids in the binding pocket of FBPase, which might be significant for the inhibitory activity of these derivatives against FBPase. Further modifications to the *N*-arylsulfonyl-indole-2-carboxamide scaffold were necessary, and the concluded SARs could provide some guidance for the design of novel FBPIs.

Three hit compounds (**VS01**, **VS02**, and **VS03**) were obtained by systematical virtual screening methods, including pharmacophore- and docking-based screenings as well as ADME predictions. The docking results indicated that **VS01**, **VS02**, and **VS03** interacted with FBPase via a similar pattern with compound **75**. The amino residues Met18, Leu30, and Thr31 were crucial for the hydrogen-bond formations with FBPIs, and Gly21 and Leu30 were essential for the hydrophobic interactions with the aromatic rings of FBPIs. The MD simulations indicated that **VS01** and **VS02** could bind stably to the receptor without significant conformational changes in the binding pocket during the dynamic simulation. **VS01** had the most potential to be a highly active inhibitor in the screened compounds due to its lowest binding energies as calculated. These screened compounds remain to be synthesized and examined for their inhibitory activity against FBPase. We expect that these results will be helpful for the rational design of novel FBPIs, and the screened hit compounds might be useful in the further development of novel FBPIs.

## Figures and Tables

**Table 1:** Statistical results of the constructed 3D-QSAR models.

	Model	*q* ^2^	ONC	SEE	*R* ^2^	*F*	Field Contributions (%)				
							S	E	H	D	A
CoMFA	S + E	0.709	8	0.097	0.979	391.215	0.720	0.280			
CoMSIA	S + E + D + A	0.716	9	0.100	0.978	255.130	0.210	0.453		0.164	0.173
	S + E + H + D + A	0.688	7	0.137	0.957	169.816	0.174	0.396	0.159	0.164	0.107
	S + E + H + D	0.680	8	0.108	0.974	242.510	0.191	0.444	0.200		0.165
	S + H + D + A	0.674	10	0.127	0.965	129.665	0.310		0.292	0.231	0.167
	E + H + D + A	0.656	7	0.151	0.948	139.441		0.482	0.194	0.200	0.165
	S + E + H + A	0.645	8	0.111	0.972	229.649	0.207	0.472	0.201	0.120	
	S + E + D	0.709	8	0.125	0.965	197.980	0.250	0.572		0.178	
	S + E + A	0.684	8	0.109	0.974	240.297	0.251	0.551			0.198
	E + H + D	0.647	9	0.106	0.975	224.324		0.543	0.268	0.189	
	S + E + H	0.632	7	0.132	0.961	185.138	0.233	0.563	0.204		
	S + H + A	0.628	9	0.144	0.955	120.028	0.404		0.362		0.234
	S + H + D	0.608	8	0.114	0.940	101.829	0.374		0.322	0.303	
	E + D	0.702	8	0.157	0.945	111.999		0.779		0.221	
	S + E	0.682	6	0.171	0.932	123.398	0.284	0.716			

*q*^2^: Cross-validated correlation coefficient; ONC: Optimal number of components; SEE: Standard error of estimate; *R*^2^: Non-cross-validated correlation coefficient; *F*: *F*-statistic values.

**Table 2:** External validation parameters of the 3D-QSAR models.

Parameters	*r_pre_* ^2^	*k*	*k*′	$\frac{r^{2}-r_{0}^{\;\;2}}{r^{2}}\;$	$\frac{r^{2}-r_{0}^{\;\;\prime 2}}{r^{2}}\;$	*r_m_* ^2^	*r_m_* *′* ^2^	$\bar{r_{m}^{\;\;2}}$	RMSE	MAE
CoMFA	0.932	0.994	1.006	0.001	0.003	0.901	0.879	0.890	0.136	0.227
CoMSIA	0.890	0.996	1.003	0.051	0.012	0.720	0.821	0.770	0.173	0.223

*r_pre_*^2^:Predictive correlation coefficient; *k* (predicted vs. actual activities) and *k′* (actual vs. predicted activities): The slope of regression lines with a zero intercept; *r*^2^: The regression line coefficient of correlation for the test set compounds; *r_0_*^2^ (predicted vs. actual activities) and *r_0_′*^2^ (actual vs. predicted activities): The correlation coefficient of regression lines with a zero intercept; *r_m_*^2^: Calculated by [*r*^2^ (1*−*(*r*^2^ *−* *r_0_*^2^)^1/2^)]; *r_m_′*^2^: Calculated by [*r*^2^ (1*−*(*r*^2^ *−* *r_0_′*^2^)^1/2^)]; $\bar{r_{m}^{\;2}}$: The average value of *r_m_*^2^ and *r_m_′*^2^; RMSE: Root mean square error for the test set compounds; MAE: Mean absolute error for the test set compounds.

**Table 3:** Statistical results of the ten top-scoring pharmacophore models.

Name	SPECIFICITY	N_HITS	FEATS	PARETO	ENERGY	STERICS	HBOND	MOL_QRY
MODEL_01	3.439	7	10	0	375.51	2500.6	730.9	213.08
MODEL_02	4.480	5	9	0	1066.94	2531.6	729.7	253.40
MODEL_03	3.410	7	9	0	31.81	2210.7	694.3	146.23
MODEL_04	4.327	8	9	0	68.89	2241.9	716.2	184.60
MODEL_05	4.113	7	10	0	40.75	2092	694.8	210.56
MODEL_06	3.475	5	10	0	57.5	2456.3	688.6	195.00
MODEL_07	4.661	8	9	0	210.92	2459.1	728.9	171.08
MODEL_08	4.001	6	12	0	714.62	2523.8	652.8	296.56
MODEL_09	4.328	7	9	0	487.87	2358	734.5	172.41
MODEL_10	4.681	7	8	0	173.61	2459.1	716.6	107.49

**Table 4:** Chemical structures and docking scores of compounds **75**, **86**, and the screened hits (**VS01**, **VS02**, and **VS03**).

No.	Structure	Total Score
**75**	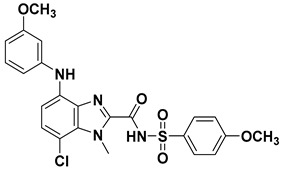	7.08
**86**	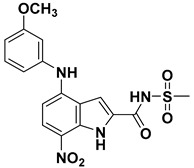	4.74
**VS01**	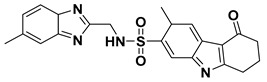	7.85
**VS02**	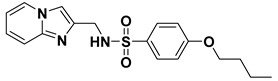	6.98
**VS03**	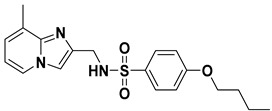	6.12

**Table 5:** Binding free energies (kJ/mol) of the screened hit compounds (**VS01** and **VS02**) in FBPase using compounds **75** and **86** as reference molecules.

System	FBPase-75	FBPase-86	FBPase-VS01	FBPase-VS02
van der Waal energy	−143.12 ± 7.43	−74.52 ± 4.41	−181.10 ± 5.94	−145.09 ± 12.29
Electrostatic energy	−68.41 ± 8.80	−34.62 ± 5.47	−61.10 ± 9.40	−46.80 ± 13.98
Polar solvation energy	145.28 ± 10.45	81.69 ± 7.72	153.16 ± 13.12	111.41 ± 23.14
SASA energy	−16.49 ± 0.96	−15.42 ± 3.13	−18.38 ± 0.99	−16.92 ± 0.83
Binding free energy	−82.73 ± 8.60	−42.87 ± 6.31	−107.42 ± 10.48	−97.40 ± 16.10

## Data Availability

Data is contained within the article and Supplementary Materials.

## Supplementary Materials

The supporting information can be downloaded at: https://www.mdpi.com/article/10.3390/ijms231810259/s1.
