# Supplementary material for: Exploration of N-Arylsulfonyl-indole-2-carboxamide Derivatives as Novel Fructose-1,6-bisphosphatase Inhibitors by Molecular Simulation
Source: Int J Mol Sci. 2022 Sep 6;23(18):10259. doi: 10.3390/ijms231810259 (PMC9499002; doi:10.3390/ijms231810259)
Supplement: Supplementary file 1 [file ijms-23-10259-s001.zip › ijms-1890847-supplementary.pdf]

Supplementary materials

Exploration of *N*-arylsulfonyl-indole-2-carboxamide derivatives as novel fructose-1,6-bisphosphatase inhibitors by molecular simulation

Yilan Zhao [a], Honghao Yang [a], Fengshou Wu [a], Xiaogang Luo [a, b, c],
Qi Sun [a, c], Weiliang Feng [a] *, Xiulian Ju [a], Genyan Liu [a, c] *

[a] *Hubei Key Laboratory of Novel Reactor and Green Chemical Technology, Key Laboratory for Green Chemical Process of Ministry of Education, School of Chemical Engineering and Pharmacy, Wuhan Institute of Technology, Wuhan 430205, P. R. China*

[b] *School of Materials Science and Engineering, Zhengzhou University, No. 100 Science Avenue, Zhengzhou City, 450001, Henan Province, P. R. China*

[c] *Key Laboratory of Novel Biomass-Based Environmental and Energy Materials in Petroleum and Chemical Industry, Wuhan Institute of Technology, Wuhan 430205, P. R. China*

*Corresponding authors: Genyan Liu, Weiliang Feng

E-mail addresses: liugenyan@wit.edu.com (G. Liu); fengweiliangax@126.com

Table S1. Chemical structures and the experimental activity of the compounds as FBPIs.

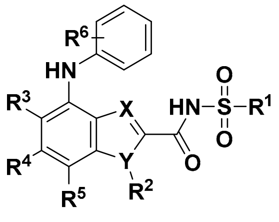

1-73

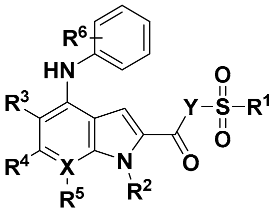

74-75

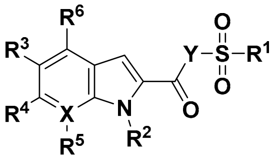

76-85

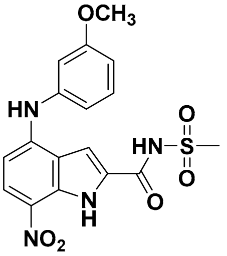

86

No.	X	Y	R^1	R^2	R^3	R^4	R^5	R^6	pIC_{50}	Predicted pIC_{50}
**1**	C	N	cPr	H	H	H	NO_2	2-OMe	5.538	5.637
**2**	C	N	Ph	H	H	H	NO_2	2-OMe	6.854	6.801
**3**	C	N	3-methoxyphenyl	H	H	H	NO_2	2-OMe	6.824	6.861
**4#**	C	N	4- methoxyphenyl	H	H	H	NO_2	2-OMe	6.721	6.618
**5**	C	N	2-fluorophenyl	H	H	H	NO_2	2-OMe	6.620	6.659
**6**	C	N	3-fluorophenyl	H	H	H	NO_2	2-OMe	6.854	6.827
**7**	C	N	4-fluorophenyl	H	H	H	NO_2	2-OMe	6.796	6.889
**8**	C	N	3-nitrophenyl	H	H	H	NO_2	2-OMe	7.000	6.962
**9**	C	N	4-nitrophenyl	H	H	H	NO_2	2-OMe	6.678	6.541
**10**	C	N	4-(trifluoromethoxy)phenyl	H	H	H	NO_2	2-OMe	6.659	6.642
**11**	C	N	thiophen-2-yl	H	H	H	NO_2	2-OMe	6.495	6.449
**12#**	C	N	naphthalen-2-yl	H	H	H	NO_2	2-OMe	6.553	6.300
**13**	C	N	Ph	H	H	H	NO_2	2-OMe	5.509	5.577
**14**	C	N	Ph	H	H	H	NO_2	4-OMe	6.000	5.963
**15#**	C	N	3-methoxyphenyl	H	H	H	NO_2	3-OEt	6.921	6.882
**16#**	C	N	3-methoxyphenyl	H	H	H	NO_2	3-OCF_2H	6.886	7.193
**17**	C	N	3-methoxyphenyl	H	H	H	NO_2	3-Me	6.824	6.649
**18**	C	N	3-methoxyphenyl	H	H	H	NO_2	3-Et	6.367	6.483
**19**	C	N	3-methoxyphenyl	H	H	H	NO_2	3-acetamido	6.796	6.826
**20**	C	N	3-methoxyphenyl	H	H	H	NO_2	3-F	5.996	6.121
**21**	C	N	3-methoxyphenyl	H	H	H	NO_2	4-F	5.821	5.879
**22**	C	N	3-methoxyphenyl	H	H	H	NO_2	3-CF_3	6.180	6.131
**23**	C	N	3-methoxyphenyl	H	H	H	NO_2	4-CF_3	6.041	6.000
**24#**	C	N	3-methoxyphenyl	H	H	H	NO_2	3-NO_2	6.854	7.100
**25**	C	N	3-methoxyphenyl	H	H	H	NO_2	4-NO_2	6.456	6.449
**26**	C	N	3-methoxyphenyl	H	H	H	NO_2	3-morpholino	6.509	6.557
**27#**	C	N	3-methoxyphenyl	H	H	H	NO_2	4- morpholino	5.963	6.007
**28#**	C	N	3-methoxyphenyl	H	H	H	NO_2	3,5-dimethoxy	6.854	6.857
**29**	C	N	3-methoxyphenyl	H	H	H	NO_2	2,3- dimethoxy	5.836	5.794
**30**	C	N	3-methoxyphenyl	H	H	H	NO_2	2,4- dimethoxy	5.893	5.962

Table S1. (Continued)

**31#**	C	N	3-methoxyphenyl	H	H	H	NO_2	4-Cl-3-OMe	6.921	6.915
**32**	C	N	3-methoxyphenyl	H	H	H	NO_2	4-F-3-OMe	6.398	6.333
**33**	C	N	3-methoxyphenyl	H	H	H	NO_2	3,4,5-triomethoxy	6.699	6.779
**34**	C	N	3-methoxyphenyl	H	F	H	NO_2	2-OMe	6.585	6.433
**35**	C	N	3-methoxyphenyl	H	Cl	H	NO_2	2-OMe	6.022	6.151
**36**	C	N	3-methoxyphenyl	H	Me	H	NO_2	2-OMe	6.022	5.896
**37**	C	N	3-methoxyphenyl	H	H	H	H	2-OMe	5.432	5.629
**38#**	C	N	3-methoxyphenyl	H	F	H	H	2-OMe	5.921	5.704
**39**	C	N	3-methoxyphenyl	H	Cl	H	NO_2	2-OMe	5.959	5.647
**40**	C	N	3-methoxyphenyl	H	Me	H	NO_2	2-OMe	5.444	5.512
**41#**	C	N	3-methoxyphenyl	H	H	F	NO_2	2-OMe	6.244	6.079
**42#**	C	N	3-methoxyphenyl	H	H	Cl	NO_2	2-OMe	6.796	7.102
**43#**	C	N	3-methoxyphenyl	H	H	CN	NO_2	2-OMe	6.444	6.538
**44#**	C	N	3-methoxyphenyl	H	H	CF_3	NO_2	2-OMe	6.292	6.137
**45#**	C	N	3-methoxyphenyl	Me	H	H	NO_2	3-OMe	7.398	7.181
**46**	C	N	3-methoxyphenyl	Et	H	H	NO_2	3-OMe	6.678	6.700
**47**	C	N	3-methoxyphenyl	iBu	H	H	NO_2	3-OMe	6.398	6.341
**48**	C	N	3-methoxyphenyl	Me	H	H	NO_2	3-Me	7.046	7.179
**49#**	C	N	3-methoxyphenyl	Me	H	H	NO_2	3-EtO	6.638	6.951
**50**	C	N	3-methoxyphenyl	Me	H	H	NO_2	3-acetamido	7.229	7.136
**51#**	C	N	3-methoxyphenyl	Me	H	H	NO_2	3,5-dimethoxy	7.200	7.373
**52#**	C	N	3-methoxyphenyl	Me	H	H	NO_2	3,4,5-trimethoxy	5.886	5.818
**53**	C	N	3-methoxyphenyl	Me	H	H	NO_2	4-Cl-3-OMe	7.200	7.125
**54**	C	N	4-methoxyphenyl	Me	H	H	NO_2	2-OMe	7.284	7.309
**55**	C	N	Ph	Me	H	H	Cl	2-OMe	6.745	6.810
**56**	C	N	3-methoxyphenyl	Me	H	H	Cl	2-OMe	6.824	6.817
**57**	C	N	2-fluorophenyl	Me	H	H	Cl	2-OMe	6.854	6.879
**58#**	C	N	3-fluorophenyl	Me	H	H	Cl	2-OMe	6.638	6.800
**59#**	C	N	4- fluorophenyl	Me	H	H	Cl	2-OMe	6.328	6.272
**60**	C	N	3-bromophenyl	Me	H	H	Cl	2-OMe	6.854	6.898
**61**	C	N	4-tolyl	Me	H	H	Cl	2-OMe	7.149	6.921
**62**	C	N	cyanophenyl	Me	H	H	Cl	2-OMe	7.252	7.324
**63**	C	N	3-isobutylphenyl	Me	H	H	Cl	2-OMe	6.027	6.119
**64**	C	N	pyridinyl	Me	H	H	Cl	2-OMe	6.658	6.683
**65#**	C	N	2-(trifluoromethoxy)phenyl	Me	H	H	Cl	2-OMe	6.114	6.062
**66**	C	N	3-(trifluoromethoxy)phenyl	Me	H	H	Cl	2-OMe	7.071	7.037
**67**	C	N	4-(trifluoromethoxy)phenyl	Me	H	H	Cl	2-OMe	7.155	7.047
**68#**	C	N	2-isobutylthiophene	Me	H	H	Cl	2-OMe	6.886	7.043
**69**	C	N	1-methylindoline	Me	H	H	Cl	2-OMe	6.229	6.325

Table S1. (Continued)

**70**	C	NH	3-methoxyphenyl	-	H	H	Cl	2-OMe	7.000	7.078
**71**	N	N	3-methoxyphenyl	Me	H	H	Cl	2-OMe	7.569	6.752
**72**	C	N	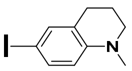	Me	H	H	Cl	2-OMe	6.268	6.235
**73**	C	N	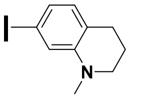	Me	H	H	Cl	2-OMe	6.174	6.146
**74**	N	NH	3-methoxyphenyl	H	H	H	-	2-OMe	6.149	6.369
**75**	C	NH	3-methoxyphenyl	Me	H	H	Cl	2-OMe	7.538	7.594
**76**	C	NH	3-methoxyphenyl	H	H	H	NO_2	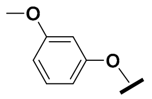	5.854	5.863
**77**	C	NH	3-methoxyphenyl	H	H	H	NO_2	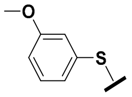	6.155	6.136
**78**	C	NH	3-methoxyphenyl	H	H	H	NO_2	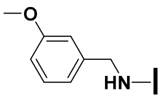	6.013	5.997
**79**	C	NH	3-methoxyphenyl	H	H	H	NO_2	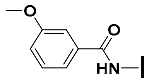	6.060	6.070
**80**	C	NH	3-methoxyphenyl	H	H	H	NO_2	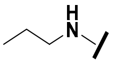	5.854	5.747
**81**	C	NH	3-methoxyphenyl	H	H	H	NO_2	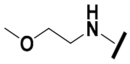	5.770	5.754
**82**	C	NH	3-methoxyphenyl	H	H	H	NO_2	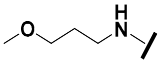	5.745	5.721
**83**	C	NH	3-methoxyphenyl	H	H	H	NO_2	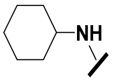	5.699	5.575
**84**	C	NH	3-methoxyphenyl	H	H	H	NO_2	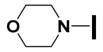	4.449	4.408
**85**	C	NH	Ph	H	H	H	NO_2	Cl	4.614	4.711
**86**	-	-	-	-	-	-	-	-	< 4.304	-

[#] The test set molecules used to validate the 3D-QSAR models.

Table S2. Chemical structures and docking scores of the twelve screened compounds.

Hit compound	Structure	Total score
ZINC32837457	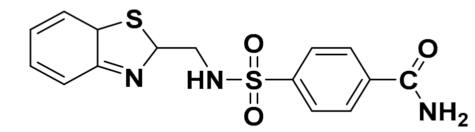	7.96
**ZINC15733809** (**VS01**)	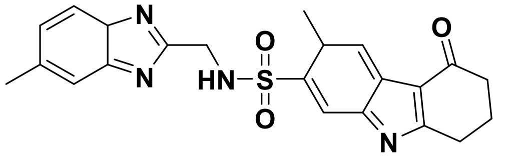	7.85
**ZINC02961023** (**VS02**)	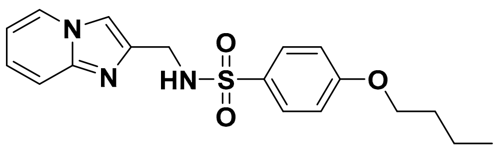	6.98
ZINC08670831	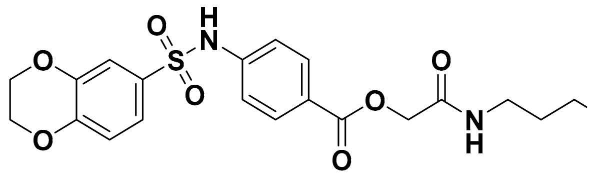	6.15
**ZINC02961075** (**VS03**)	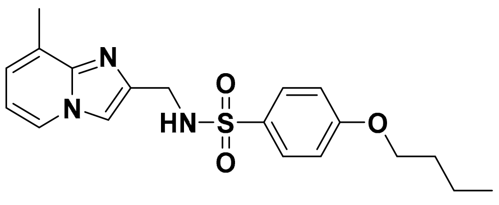	6.12
ZINC47146484	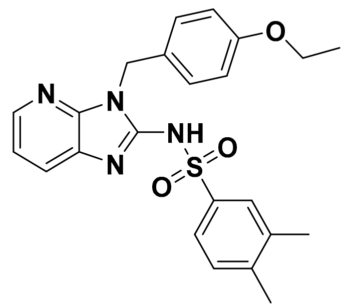	6.00
ZINC48903386	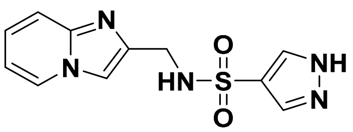	5.95
ZINC47147329	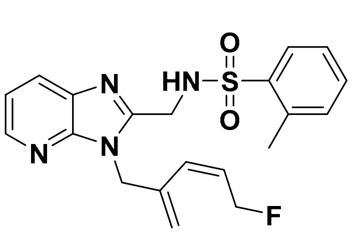	5.70

Table S2. (Continued)

ZINC47147354	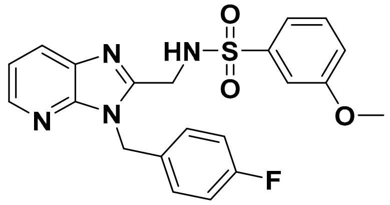	5.24
ZINC58356765	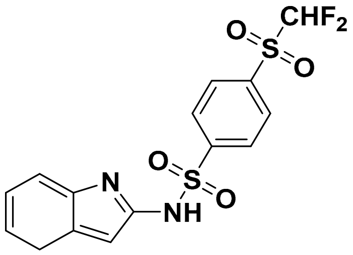	5.19
ZINC47146462	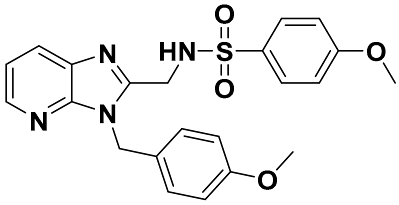	5.16
ZINC08600440	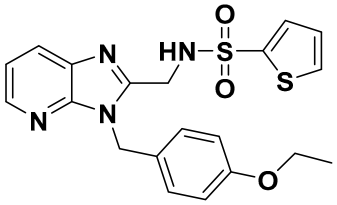	5.09

Table S3. Predicted ADME parameters and drug-like properties of compound **75** and the screened compounds.

Properties	Parameters	75	VS01	VS02	VS03
**Physiochemical Properties**	MW[a] (g/mol)	499.97	422.50	359.44	373.47
	Rotatable bonds	8	4	8	8
	H-bond acceptors	5	5	5	6
	H-bond donors	2	3	1	1
	TPSA[b] ($Å^2$)	107.04	116.09	81.08	81.08
**Lipophilicity**	iLOGP	3.44	1.73	3.20	3.28
	XLOGP3	4.85	2.97	3.48	3.84
	WLOGP	5.79	4.59	3.92	4.23
	MOLGP	2.35	1.51	1.35	1.58
	SILICOS-IT	3.02	4.50	1.98	2.51
	Consensus LogP	3.89	3.06	2.79	3.09
**Water Solubility**	ESOL Class	MS[c]	MS	MS	MS
	Ali Class	PS	MS	MS	MS
	SILICOS-IT Class	PS[d]	PS	PS	PS
**Pharmacokinetics**	GI[e] absorption	Low	High	High	High
	BBB[f] permeant	No	No	No	No
**Drug-like Properties**	Lipinski violations	0	0	0	0
	Ghose violations	3	0	0	0
	Verber violations	0	0	0	0
	Egan violations	0	0	0	0
	Muegge violations	0	0	0	0
	Bioavailability Score	0.55	0.55	0.55	0.55
**Medicinal Chemistry**	PAINS[g] alerts	0	0	0	0
	Brenk alerts	0	0	0	0
	Leadlikeness violations	3	1	2	3
	Synthetic accessibility	3.43	3.24	2.91	3.03

[a] Molecular weight. [b] Total polar surface area. [c] Moderately soluble. [d] Poorly soluble. [e] Gastrointestinal. [f] Blood-brain barrier. [g] Pan assay interference compounds.

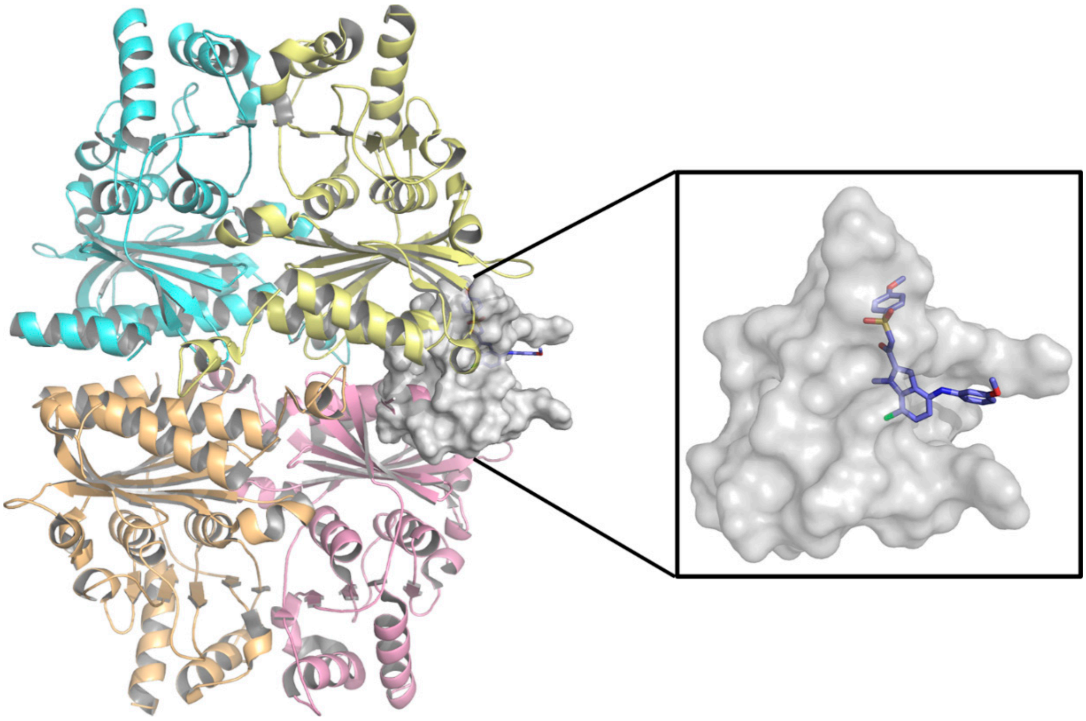

Figure S1. The 3D structure of FBPase in complex with compound **75** (PDB code: 6LW2). The AMP binding site is shown as a grey surface.

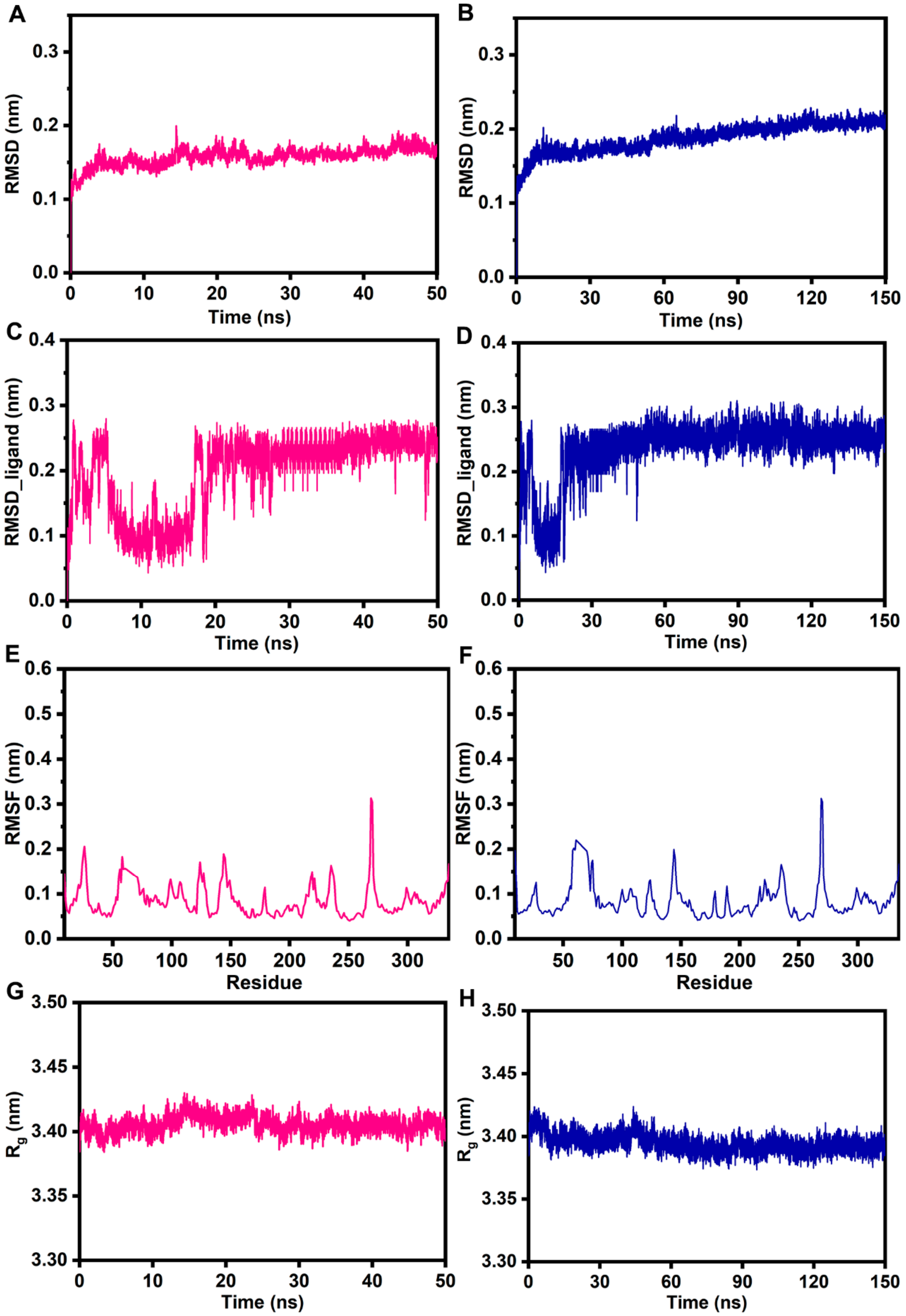

Figure S2. The MD results of the 50 ns (**A**, **C**, **E**, **G**) and 100 ns (**B**, **D**, **F**, **H**) simulations of FBPase-**75** complex: RMSD values of backbone atoms (**A**, **B**), RMSD values of ligand atoms (**C**, **D**), RMSF values of Chain A residues (**E**, **F**), and R_g values of the proteins (**G**, **H**).

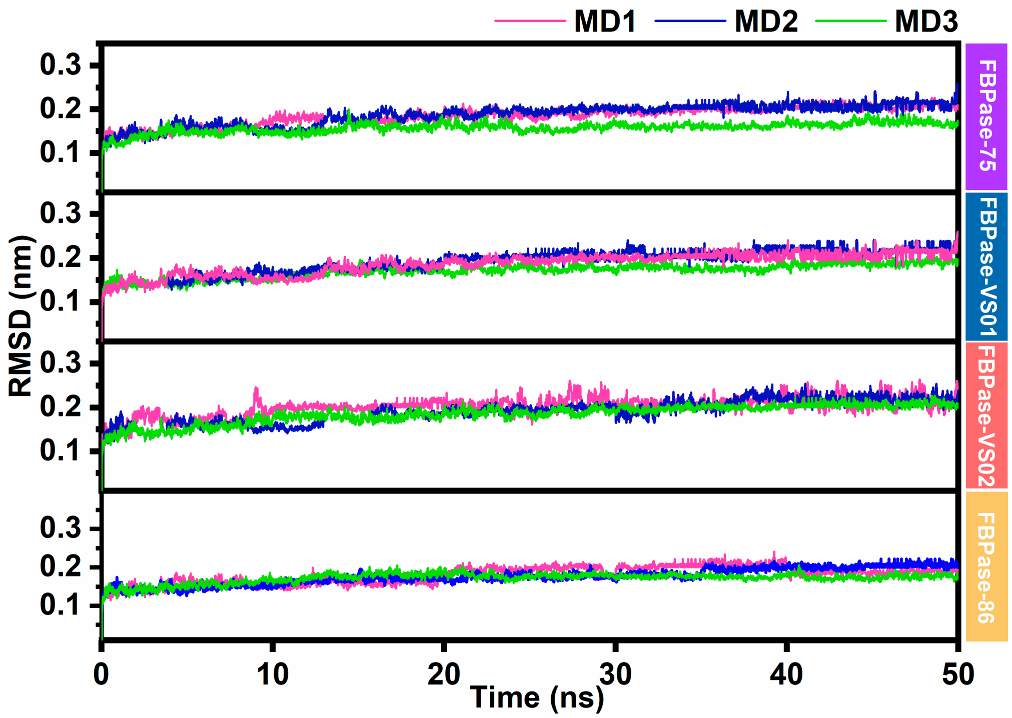

Figure S3. The RMSD plots of the protein backbones for the four complexes: FBPase-**75**, FBPase-**86**, FBPase-**VS01**, and FBPase-**VS02** derived from the triplicate MD simulations (MD1-MD3).

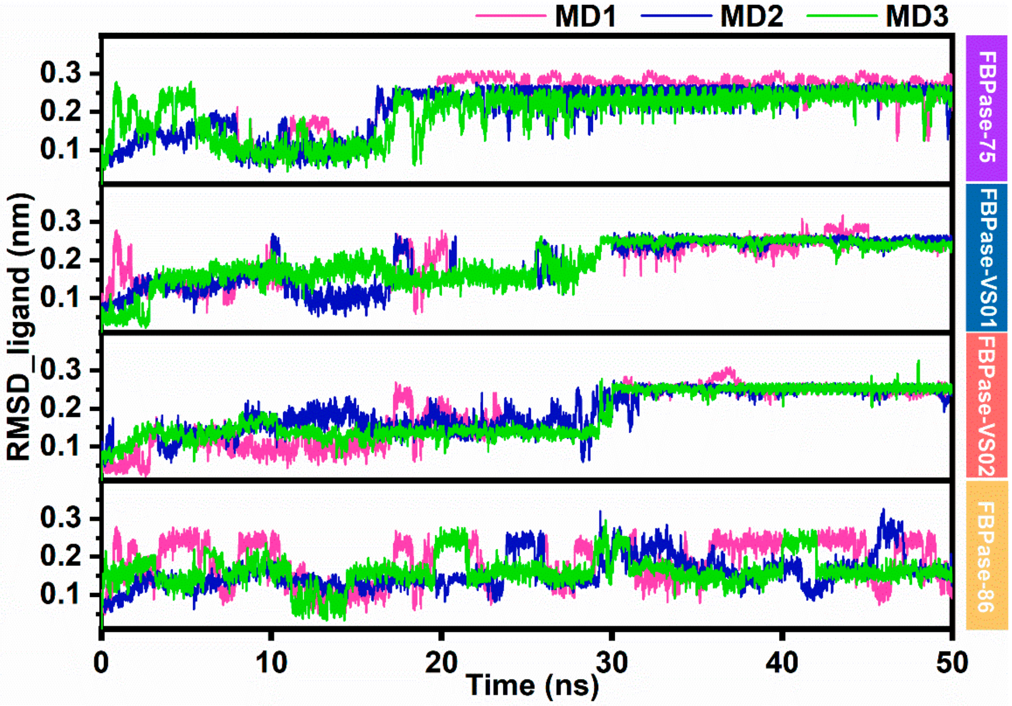

Figure S4. The RMSD plots of the ligands for the four complexes: FBPase-**75**, FBPase-**86**, FBPase-**VS01**, and FBPase-**VS02** derived from the triplicate MD simulations (MD1-MD3).

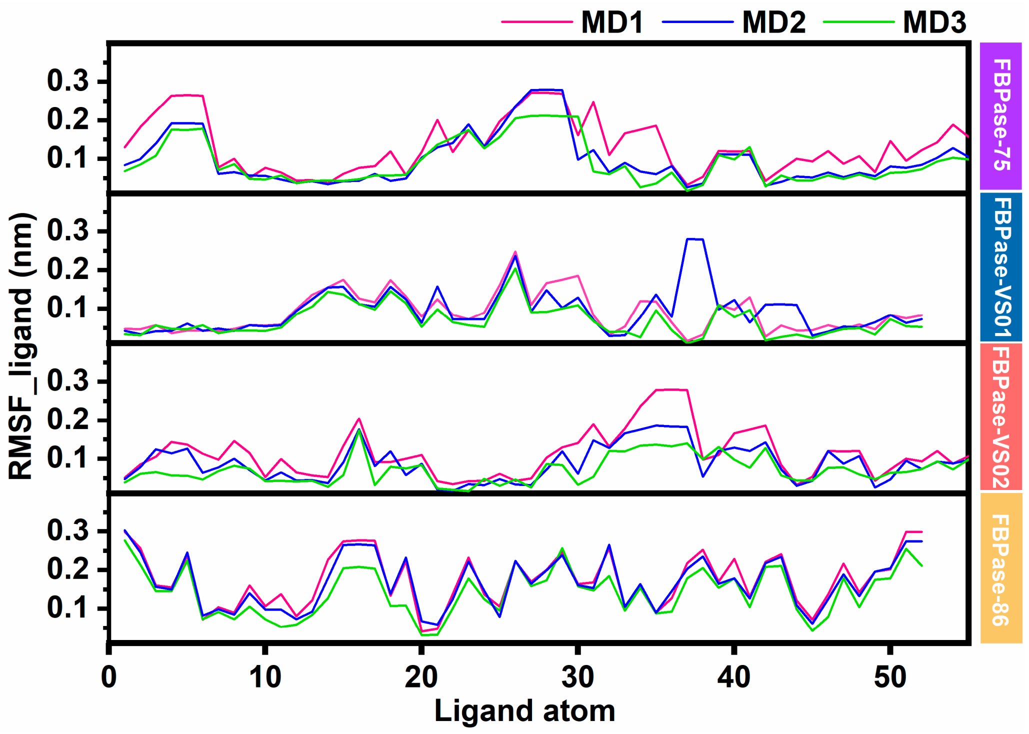

Figure S5. The RMSF plots of the ligands for the four complexes: FBPase-**75**, FBPase-**86**, FBPase-**VS01**, and FBPase-**VS02** derived from the triplicate MD simulations (MD1-MD3).

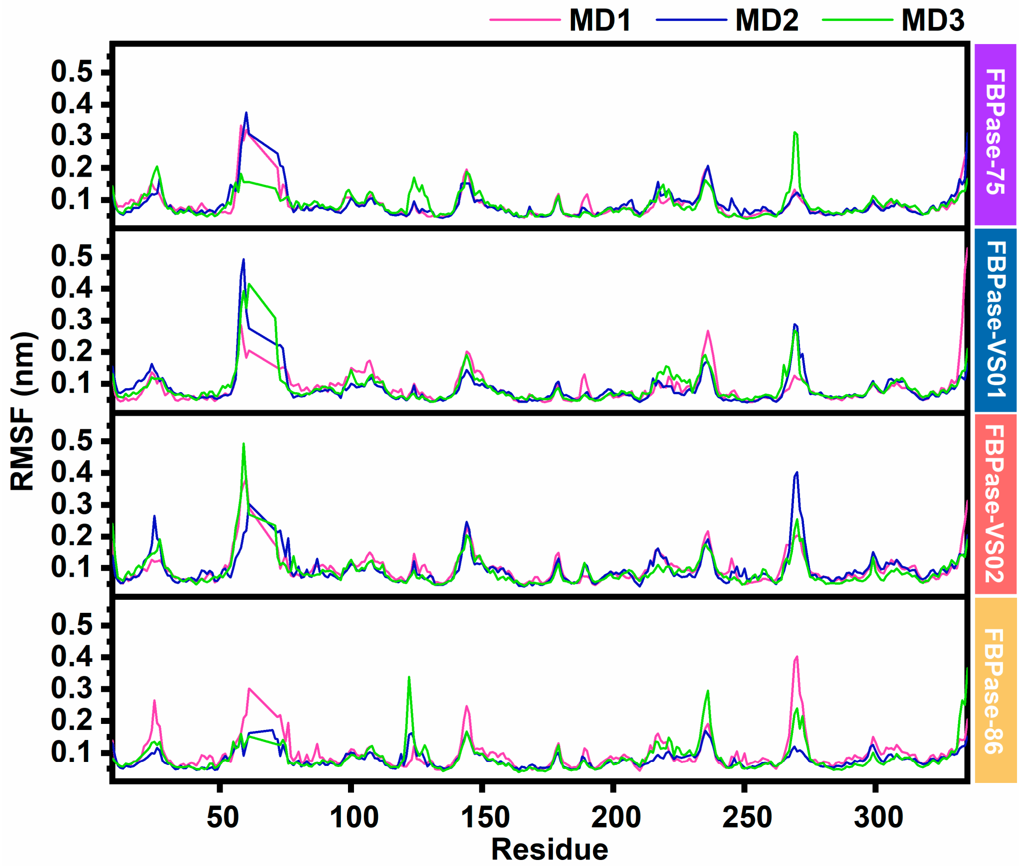

Figure S6. The RMSF plots of the Chain A residues for the four complexes: FBPase-**75**, FBPase-**86**, FBPase-**VS01**, and FBPase-**VS02** derived from the triplicate MD simulations (MD1-MD3).

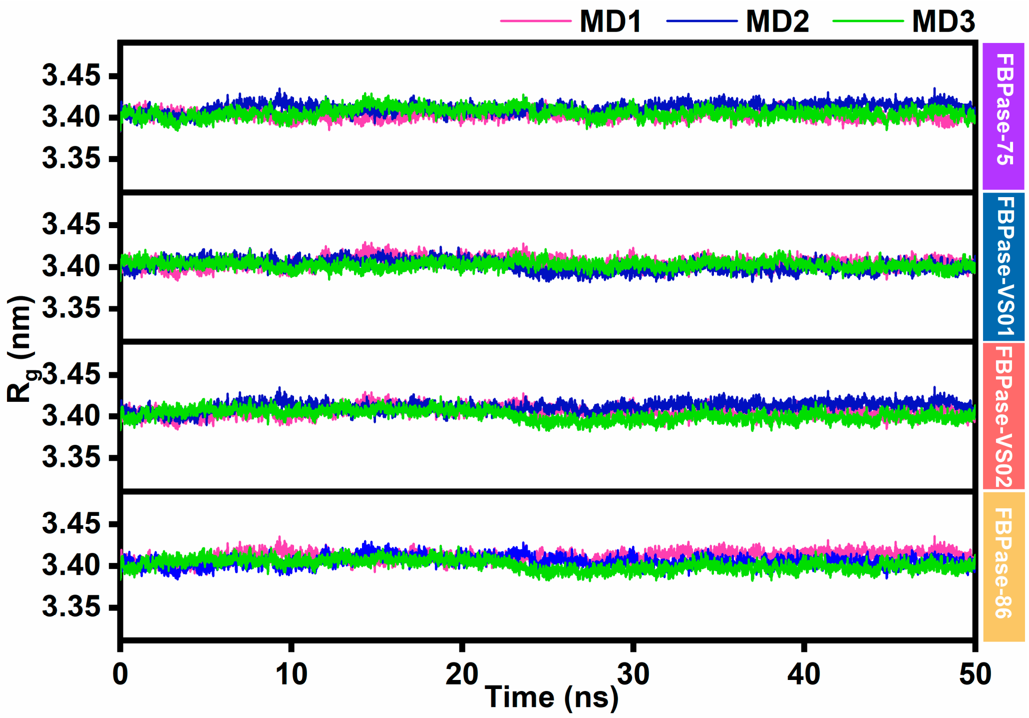

Figure S7. The R_g plots of the proteins for the four complexes: FBPase-**75**, FBPase-**86**, FBPase-**VS01**, and FBPase-**VS02** derived from the triplicate MD simulations (MD1-MD3).
